# Serum lncRNA H19/miR-675 /PPARα expression before middle gestation and their associations with macrosomia risk in singleton pregnancies without gestational diabetes mellitus: a preliminary study

**DOI:** 10.7717/peerj.20793

**Published:** 2026-02-16

**Authors:** Qiuyan Yu, Ming Min Jin, Miao Miao Ding, Bin Wei Cheng, Xiao Xia He, Xin Jun Yang

**Affiliations:** 1 Department of Epidemiology and Health Statistics, School of Public Health, Wenzhou Medical University, Wenzhou, Zhejiang, China; 2 Clinical Laboratory, The Affiliated Hospital of Inner Mongolia Medical University, Hohhot, Inner Mongolia, China

**Keywords:** Macrosomia, LncRNA H19, Peroxisome proliferator-activated receptors, Serum biomarkers, Non-gestational diabetes mellitus

## Abstract

**Background:**

The roles of maternal serum lncRNA H19, miR-675, and * PPARα* protein levels before mid-pregnancy in predicting macrosomia remain unclear. This study aimed to investigate whether the expression of these serum molecules is associated with the risk of macrosomia in singleton pregnancies without gestational diabetes mellitus.

**Methods:**

A nested case-control study was conducted within a prospective cohort study of 898 women with singleton pregnancies. Mothers of liveborn macrosomic newborns constituted the case group, and a random sample of mothers of the normal-birthweight newborns, matched on gestational age at blood collection and delivery date, served as controls. Serum levels of lncRNA H19, miR-675, * PPARα* protein, and serum lipids were measured before 20 weeks’ gestation. Logistic regression, restricted cubic spline analysis, and stratified analysis were used to assess the associations. Predictive performance was explored using area under the receiver operating characteristic curve, net reclassification index (NRI), and integrated discrimination improvement (IDI).

**Results:**

No significant differences were observed in lncRNA H19 (*Z* =  − 0.344, *P* = 0.731), miR-675 (*Z* =  − 1.376, *P* = 0.169), or * PPARα* protein levels (*Z* < 0, *P* = 0.999) between macrosomia and control groups. However, in women with pre-pregnancy BMI < 24 kg/m^2^, lower * PPARα* protein levels (tertile 2 *vs.* tertile 3) were associated with a 70% reduced risk of macrosomia (*OR* = 0.30, 95% CI [0.09–0.99], * P* = 0.049). The NRI and IDI of the combined model incorporating serum lncRNA H19, miR-675, and PPARα protein levels were statistically superior to lipid-based models (*P* < 0.05).

**Conclusions:**

Serum lncRNA H19 and miR-675 were not associated with macrosomia risk. Lower serum * PPARα* protein levels in early pregnancy may be associated with a reduced risk of macrosomia, particularly in non-obese women. The combined biomarkers demonstrated preliminary predictive potential in exploratory analysis, but validation in larger cohorts is required.

## Introduction

Macrosomia, defined in Asia as newborn birth weight ≥ 4,000 g, is associated with an increased risk of adverse pregnancy events and adverse perinatal outcomes, and metabolism disorders in later in life (Beta et al., 2019; Koyanagi et al., 2013). In China, the national prevalence of macrosomia was 16.3% between 2019 and 2024, with regional variation in which the highest reported rate reached 13.9% (Wang et al., 2018; Zhou et al., 2023). Efforts to manage gestational diabetes mellitus (GDM)—including behavioral interventions, early diagnosis, and timely treatment—have focused on reducing and controlling maternal blood glucose. However, overall prevalence of macrosomia has not decreased effectively, partly because of the rising proportion of macrosomic births among women without GDM (Liang, Zhang & Li, 2017). A recent population-based study further indicated that GDM was not significantly associated with the occurrence of large-for-gestational-age newborns, suggesting that the mechanisms underlying macrosomia remain incompletely understood (Pittyanont et al., 2024).

The lncRNA H19 is located within an imprinted regulatory region of the IGF2/H19 locus on chromosome 11 and spans approximately 2.0 kb (Nordin et al., 2014). It plays a critical role in embryonic and placental development (Adu-Gyamfi et al., 2024; Gabory, Jammes & Dandolo, 2010). Altered expression of lncRNA H19 has been documented in conditions related to aberrant fetal growth, including abnormal birthweight (Huang et al., 2020), pre-eclampsia (Ogoyama et al., 2022), and fetal growth restriction (Tsunoda et al., 2020). H19 has the unique capacity to generate a primary 23-nucleotide miRNA precursor from exon 1 for miR-675 (Cai & Cullen, 2007). The H19/miR675 axis has been linked to placental conditions such as pre-eclampsia (Gao et al., 2012), trophoblasts proliferation (Ogoyama et al., 2021), and placental endocrine capacity (Aykroyd, Tunster & Sferruzzi-Perri, 2022), suggesting that the role of placental H19 during pregnancy is mediated through miR-675.

Peroxisome proliferator-activated receptor-α (*PPAR*α), one of the three PPAR isoforms, is highly expresses in reproductive tissues and plays a key role in lipogenesis, energy metabolism (Singh, Dhar & Karmakar, 2022), and the developmental origins of adult disease (Guo et al., 2022). The lncRNA H19/miR-675 has been shown to promote extravillous trophoblast (Ogoyama et al., 2021), and regulate hepatic energy metabolism by targeting PPARα expression (Liu et al., 2019). It has also been reported to enhance cell viability and reduce cell apoptosis in models of myocardial ischemia–reperfusion injury *via PPAR*α modulation (Luo et al., 2019). Our previous work demonstrated that placentas from pregnancies complicated by macrosomia exhibited lower expression of H19 and miR-675 and higher expression of *PPAR*α is compared with those from pregnancies with normal birthweight newborn; furthermore, knockdown of H19 increased *PPAR*α expression in HTR-8/SVneo cells (Ding et al., 2021). However, whether maternal serum lncRNA H19, miR-675, and *PPAR*α are associated with risk of macrosomia remains unclear.

This study aimed to characterize maternal serum lncRNA H19, miR-675, and *PPAR*α protein and to evaluate their associations with macrosomia risk using a nested case-control design. In addition, we assessed their potential utility as blood-based biomarkers for predicting in macrosomia in pregnancies without GDM.

## Materials & Methods

### Study population

This nested case-control study was conducted within a prospective cohort of pregnant women. Participants were women with singleton pregnancies between 13 and 20^+6^ weeks’ gestation as determined by ultrasound, who attended prenatal care at four hospitals in hospitals in Wenzhou, China, between July 2020 and April 2021. Cases were mothers who delivered a liveborn with macrosomia (birthweight ≥ 4,000 g). Controls were randomly selected from mothers within the same cohort who delivered a live, term newborn with normal birthweight (ranging from 2,500 g to 3,999 g). Controls were individually matched to each case based on both gestational age at blood collection (within ±1 week) and delivery date (within ±1 week).

Inclusion criteria for all participants were: term pregnancy (37 to 42 weeks), singleton gestation, delivery without assisted technology, and a liveborn without congenital malformations. Exclusion criteria included pre-pregnancy metabolic diseases or long-term medication use, congenital or genetic disorders, and maternal blood samples collected after 20 weeks’ gestation. All eligible women were informed about the study, and provided written informed consent. Ethical approval was obtained from the Wenzhou Medical University Ethics Committee (2018035).

### The measurement of serum Lipid profile

The maternal serum concentrations of triglyceride (TG), total cholesterol (TC), high density lipoprotein cholesterol (HDL-C) and low-density lipoprotein cholesterol (LDL-C) were measured by automatic biochemical analyzer (Mindray BS480, Shenzhen, China) in the same hospital laboratory. The detection kits (oxidase methods for TG and TC, catalase removal method for HDL-C and surfactant method for LDL-C) were purchased from Mindray (Shenzhen, China).

### Collection of survey information and delivery records

Participants completed a structured questionnaire that collected data on socio-demographic characteristics (maternal age, ethnicity, height and weight, and annual household income), lifestyle before 20 weeks’ gestation (weekly exercise frequency, nighttime sleep duration, and diet based on a simplified food-frequency questionnaire), and disease history (self-reported metabolic, genetic, congenital, mental disorders). Delivery information (neonatal sex, birthweight, survival status, and delivery mode) and maternal health records during pregnancy (gestational weight gain, and complications) were retrieved from hospital electronic medical records.

### Maternal blood sample collection and preservation

Fasting venous blood samples (five ml) were collected by trained clinicians and left at room temperature (approximately 26 °C) for 20 mins. Samples were centrifuged at 3,000 rpm for 5 min, and serum aliquots were transported on dry ice to the laboratory, transferred into RNase-free cryotubes, and stored at −80 °C.

### Quantitative real-time polymerase reaction

The total RNA was extracted from maternal serum using TRIzol reagent (Invitrogen, Carlsbad, CA, USA) and then reverse transcribed into cDNA using a reverse transcription kit (Takara, Tokyo, Japan). The RNA samples, isolated with 10 µL nuclease-free water, was quantified using Multiskan GO UV/Vis Microplate Spectrophotometer (Thermo Fisher Scientific, MA, USA). RNA samples with A260/A230 ratios between 1.8 and 2.0 and A260/A280 ratios greater than 2 were selected. Formaldehyde agarose gel was used to examine the integrity of all RNA samples (28S/18S ≈ 2.0, no visible smear). Total RNA of 600 ng was reverse transcribed for 15 min at 37 °C with PrimerScript RT Reagent Kit (TaKaRa Bio Inc., Dalian, China) according to the manufacturer’s instruction. The reaction was terminated by heating 85 °C for 5 s, and then were stored at −20 °C until next day. The cDNA levels were analyzed using a CFX96 Touch quantitative real-time polymerase chain reaction (qRT-PCR) detection system (Bio-Rad, Hercules, CA, USA) with SYBR green dye (Roche, Basel, Switzerland) according to the manufacturer’s guidelines. Each sample, containing two µL of RT product, was replicated by three times in a MicroAmp Fast Optical 96-well plate (Bio-Rad, Hercules, CA, USA) in a reaction volume (10 µL) of 2X Universal SYBR Green Fast qPCR Mix (Abclonal, Wuhan, China), and the real-time PCR was conducted with an initial denaturation of 3 min at 95 °C accompanied by 44 cycles of 5 s at 95 °C, 15 s at 53 °C, 15 s at 72 °C. Reverse transcription of miR-675 and miR-39-3p were conducted with stem-loop primers (Ribobio, Guangzhou, China). MiR-39-3p (Ribobio, Guangzhou, China) was used as the external reference for lncRNA H19 and miR-675, and standardized values were calculated. The primers of lncRNA H19 (141bp) was synthesized by Sangon (Shanghai, China). The primer sequences of lncRNA H19: Forward, 5′-GGACGTGACAAGCAGGACAT-3′; Reverse, 5′-CATAGTGCCGACTCCGTG-3′. The threshold cycles (Ct) were calculated using the CFX Maestro software. Reactions for each sample prepared technical duplicates and blank negative controls, and the relative expression was calculated by the equation 2-ΔΔCt. Variability was defined by the standard deviation (SD) of mean Cq values and low SD values corresponded to stable miR-675 or lncRNA H19. As this was a screening study, not all MIQE (Minimum Information for Publication of Quantitative Real-Time PCR Experiments) specifications were provided. All Real-time PCR experiments were compliant with the MIQE guidelines.

### Enzyme-linked immunosorbent assay

The serum was thawed at 4 °C after being removed from the −80 °C refrigerator. The concentration of the *PPAR*α protein in maternal serum was measured using enzyme-linked immunosorbent assay kits (Cusabio, Wuhan, China) according to the manufacturer’s instructions.

### Statistical analysis

Analyses were conducted in R software version 4.1.3. Questionnaire data were double-checked and entered into EpiData 3.1. Multiple imputation was applied to covariates with <30% missing data. Normally distributed continuous variables were presented as mean ± standard deviation (SD) and compared using unpaired or paired *t* test. Skewed continuous variables were expressed as median (P25, P75), and compared using the Wilcoxon signed-rank test or Mann–Whitney U test. Categorical variables were summarized as counts (%), and compared using the McNemar test, chi square test, or Fisher’s exact test. A two-tailed *P* value < 0.05 indicated statistical significance.

Correlation analyses were performed to assess the relationships among maternal serum lipids, lncRNA H19, miR-675, and *PPAR*α protein levels. Pearson’s correlation was used for variables that followed a bivariate normal distribution; otherwise, Spearman’s rank correlation was applied. Conditional multivariate logistic regression was used to estimate Odds Ratios (*ORs*) between lncRNA H19, miR-675, *PPAR*α and macrosomia, along with the corresponding 95% confidential intervals (CIs). Restricted cubic spline (RCS) based on logistic regression was performed, and the expression value of the first quartile of lncRNA H19, the third quartile of miR-675, and the first quartile of *PPAR*α at *OR* = 1 was treated as the references. Subgroup analyses were performed to evaluate the influences of three molecular indexes (lncRNA H19, miR-675, *PPAR*α) on the occurrence of macrosomia in various subgroups, including overweight before pregnancy (yes/no), parity (primiparous/multiparous), and maternal age (</≥ 30 years). Sensitivity analysis was performed in pregnant women who delivered ≤ 40 weeks’ gestation, or who were without history of macrosomia, or whose blood samples collected ≤ 13 weeks’ gestation.

Receiver operating characteristics (ROC) curves were used to evaluate the predictive performance of each of three molecular indexes (lncRNA H19, miR-675, *PPAR*α) and combined on the occurrence of macrosomia, quantified using area under curve (AUC). The net reclassification improvement (NRI) and integrated discrimination improvement (IDI) were calculated to assess incremental predictive performance beyond the basic models.

## Results

### Participant characteristics

Maternal and neonatal characteristics in the macrosomia group (*n* = 47) and matched control group are presented in Table 1, and the screening process is shown in Fig. S1. The corresponding prospective cohort of 898 participants summarized in Table S1, imputed data before and after processing are presented in Table S2. The overall prevalence of macrosomia was 6.24%. The mean birthweight of newborns with macrosomia was 4,103.43 ± 141.77 g, compared with 3,358.40 ± 288.94 g in the control group.

**Table 1:** Maternal and neonatal characteristics in the macrosomia group and the control group.

Characteristics	Macrosomia (*n* = 47)	Control (*n* = 47)	*P* values
Maternal age (years)	27.00 (24.50, 30.50)	26.00 (25.00, 28.50)	0.320
Han ethnicity	44 (93.6)	41 (87.2)	0.486
Primipara	16 (34.0)	18 (38.3)	0.830
Receiving ≤ 12 years’ education	29 (61.7)	30 (63.8)	1.000
BMI before pregnancy (kg/m^2^)	23.07 (20.68, 25.51)	20.55 (18.83, 22.25)	<0.001
Gestational age (weeks)	40.00 (39.00, 40.00)	39.00 (38.00, 40.00)	0.001
Gestational weight gain (kg)	16.50 (13.25, 19.40)	15.50 (13.25, 18.15)	0.414
Vaginal delivery	25 (53.2)	29 (61.7)	0.531
<7,000 RMB of family income per month	24 (51.1)	26 (55.3)	0.836
Working during pregnancy	19 (40.4)	23 (48.9)	0.534
<3 frequencies of physical exercise during pregnancy per week	25 (53.2)	30 (63.8)	0.834
Sleep duration during pregnancy	8.00 (8.00, 10.00)	8.00 (8.00, 9.00)	0.516
**Maternal dietary information before 20 weeks’ gestation**			
Drinking before pregnancy	4 (8.5)	1 (2.1)	0.361
Smoking before pregnancy	1 (2.1)	0 (0.0)	1.000
>7 frequencies of cereals intake per week	46 (97.9)	45 (95.7)	1.000
>7 frequencies of nuts intake per week	11 (23.4)	10 (21.3)	1.000
>7 frequencies of meats intake per week	18 (38.3)	23 (48.9)	0.405
>7 frequencies of seafood intake per week	12 (25.5)	17 (36.2)	0.372
>7 frequencies of soybean intake per week	12 (25.5)	14 (29.8)	0.818
>7 frequencies of vegetable intake per week	39 (83.0)	35 (74.5)	0.450
>1 egg per day	16 (34.0)	26 (55.3)	0.062
>7 frequencies of fruits intake per week	38 (80.9)	34 (72.3)	0.465
>7 frequencies of milk and milk products intake per week	18 (38.3)	18 (38.3)	1.000
>7 frequencies of folic acid intake per week	42 (89.4)	43 (91.5)	1.000
Having gestational hypertension	2 (4.3)	1 (2.1)	1.000
**Maternal laboratory test before 20 weeks’ gestation**			
Blood collection time (weeks)^a^	11.00 (10.00, 13.00)	11.00 (9.50, 13.00)	0.847
LncRNA H19	0.73 (0.08, 3.57)	0.75 (0.18, 3.28)	0.835
miR675	0.93 (0.16, 2.04)	1.11 (0.15, 3.32)	0.346
PPARα protein level (ng/ml)	1.20 (0.78, 1.52)	1.04 (0.79, 1.46)	0.691
TG (mmol/L)	1.69 (0.72)	1.48 (0.68)	0.160
TC (mmol/L)	4.61 (1.27)	4.55 (0.92)	0.819
HDL (mmol/L)	1.65 (0.54)	1.80 (0.39)	0.144
LDL (mmol/L)	2.57 (0.92)	2.39 (0.69)	0.303
Fasting blood glucose (mmol/L)	4.80 (4.64, 5.08)	4.65 (4.41, 4.84)	0.008
**Newborn information**			
Girls	15 (31.9)	19 (40.4)	0.520
Birthweight (g)	4,170.43 (141.77)	3,358.40 (288.94)	<0.001
Newborn length (cm)	51.00 (50.00, 52.00)	50.00 (50.00, 50.00)	<0.001

**Notes.**

Data are show as n (%), mean (SD), or median (lower quartile, upper quartile).

SD standard deviance

### Expression levels of lncRNA H19, miR-675, *PPAR*α protein level in maternal serum

**Figure 1:**
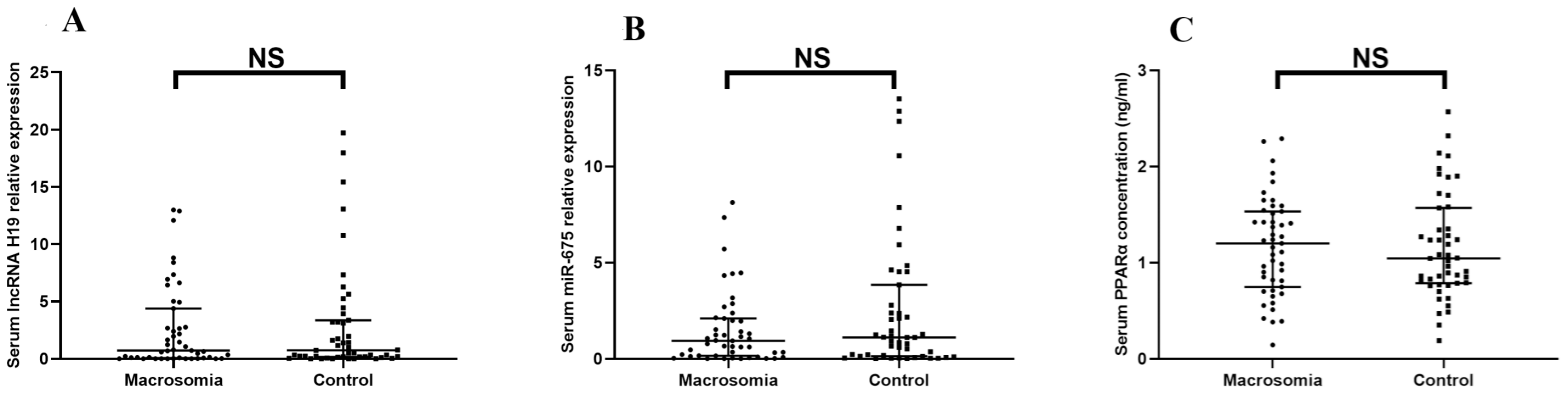
The expression of lncRNA H19, miR-675, the protein level of PPAR a in maternal serum. (A) lncRNA H19; (B) miR-675; (C) PPAR a protein levels. NS: *P* > 0.05.

There was no statistically significant difference in lncRNA H19 (Z = −0.344, *P* = 0.731) or miR-675 (Z = −1.376, *P* = 0.169) between the two groups (Figs. 1A–1B). Likewise, the serum protein expression level of *PPAR*α did not differ significantly between groups (*Z* < 0, *P* = 0.999) (Fig. 1C).

### Correlation analysis of serum lipids with serum lncRNA H19, miR-675, and *PPAR*α protein level

**Figure 2:**
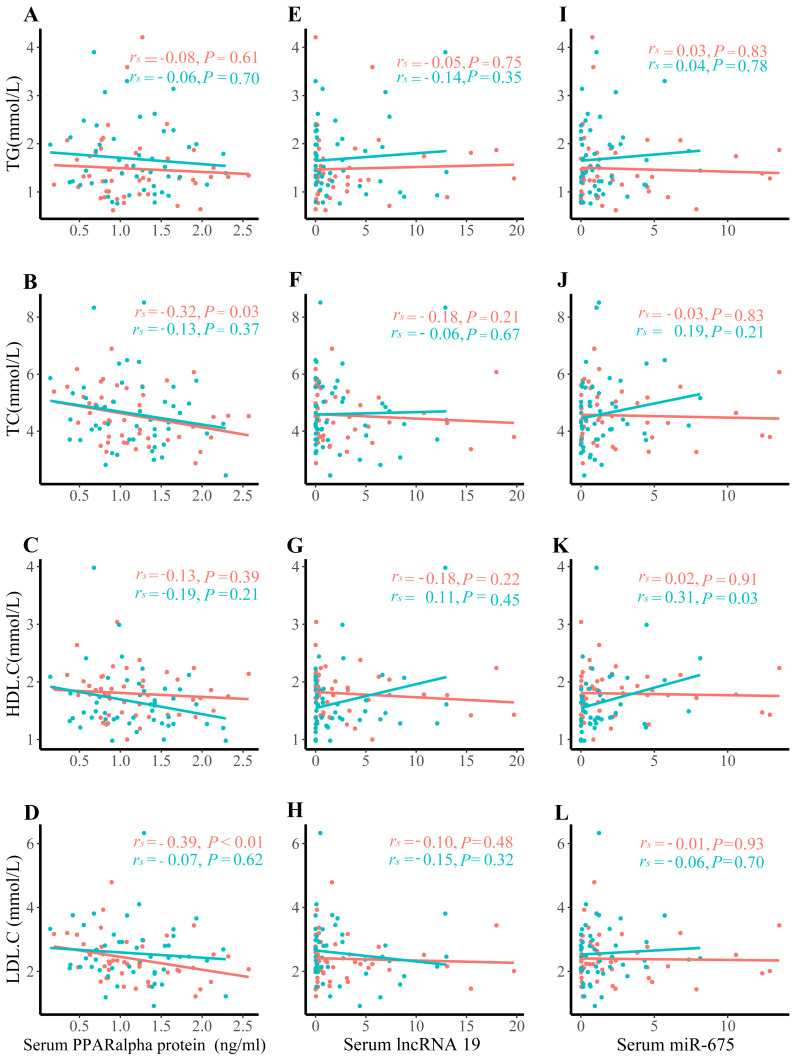
Correlation analysis between serum lipids and lncRNA H19, miR-675, and PPAR a protein expression. (A) TG and PPAR a protein expression; (B) TC and PPAR a protein expression; (C) HDL. C and PPAR a protein expression; (D) LDL.C and PPAR a protein expression; (E) TG and lncRNA H19; (F) TC and lncRNA H19; (G) HDL.C and lncRNA H19; (H) LDL.C and lncRNA H19; (I) TG and miR-675; (J) TC and miR-675; (K) HDL.C and miR-675; (L) LDL.C and miR-675; The green dots denote the macrosomia group. the red dots denote the control group.

Positive correlations were observed between serum *PPAR*α protein levels and TC (*r*_*s*_ =  − 0.32, *P* = 0.03), and between serum *PPAR*α protein level and LDL-C only in control group (Figs. 2B–2D). No associations were detected between *PPAR*α and TG, *PPAR*α and HDL-C, serum lipids and lncRNA H19, or serum lipids and miR-675 (Figs. 2E–2L).

### Associations of serum lncRNA H19, miR-675, and PPARα protein level with macrosomia

The RCS analyses indicated no correlation between lncRNA H19, miR-675, or *PPAR*α protein levels and the occurrence of macrosomia (Fig. S2). As shown in Table 2, maternal serum lncRNA H19 and miR-675 were not associated with the risk of macrosomia. Compared with the tertile 3 of *PPAR*α protein levels, tertile 2 was associated with a lower risk of macrosomia (adjusted OR: 0.15; 95% CI [0.03–0.87]), after adjustment for gestational age, pre-pregnancy BMI, and maternal age.

**Table 2:** Association of serum lncRNA H19, miR-675, and PPAR a protein levels with incidence of macrosomia. OR (95% CI) was estimated by multivariate conditional logistic regression Model 2 was conducted with adjusting for gestation in weeks, pre-pregnancy BMI, maternal age. Model 3 was conducted with adjusting for gestation in weeks, pre-pregnancy BMI, maternal age, and neonatal sex.

**Characteristics**	**Maternal serum level**			
	**No. of macrosomia/total**	**Model 1** *** cOR*** ** (95%** ***CI*** **)**	**Model 2 a*OR* (95%*CI***)	**Model 3 a*OR* (95%*CI***)
**Association of IncRNA H19 with macrosomia**				
**Per unit increase**		0.93 (0.73, 2.12)	1.29 (0.59, 2.82)	1.38 (0.56, 3.36)
Tertile 1	17/31	Reference	Reference	Reference
Tertile 2	14/32	0.70 (0.29, 1.72)	0.55 (0.13, 2.42)	0.70 (0.14, 3.40)
Tertile 3	16/31	0.91 (0.32, 2.57)	1.56 (0.33, 7.42)	1.74 (0.30, 10.05)
**Association of miR-675 with macrosomia**				
**Per unit decrease**		1.24 (0.73, 2.12)	0.93 (0.45, 1.90)	0.87 (0.40, 1.90)
Tertile 1	16/31	1.55 (0.53, 4.55)	0.80 (0.18, 3.51)	0.72 (0.15, 3.51)
Tertile 2	18/32	1.88 (0.65, 5.42)	0.50 (0.10, 2.41)	0.57 (0.11, 2.88)
Tertile 3	13/31	Reference	Reference	Reference
**Association of PPARα protein levels with macrosomia**				
**Per unit decrease**		0.70 (0.38, 1.28)	0.51 (0.21, 1.26)	0.54 (0.21, 1.40)
Tertile 1	15/31	0.42 (0.12, 1.49)	0.20 (0.03, 1.52)	0.23 (0.03, 1.83)
Tertile 2	13/32	0.33 (0.10, 1.12)	0.15 (0.03, 0.87)	0.17 (0.03, 1.01)
Tertile 3	19/31	Reference	Reference	Reference

**Notes.**

OR (95% CI) was estimated by multivariate conditional logistic regression.

Model 2 was conducted with adjusting for gestation in weeks, pre-pregnancy BMI, maternal age.

Model 3 was conducted with adjusting for gestation in weeks, pre-pregnancy BMI, maternal age, and neonatal sex.

**Figure 3:**
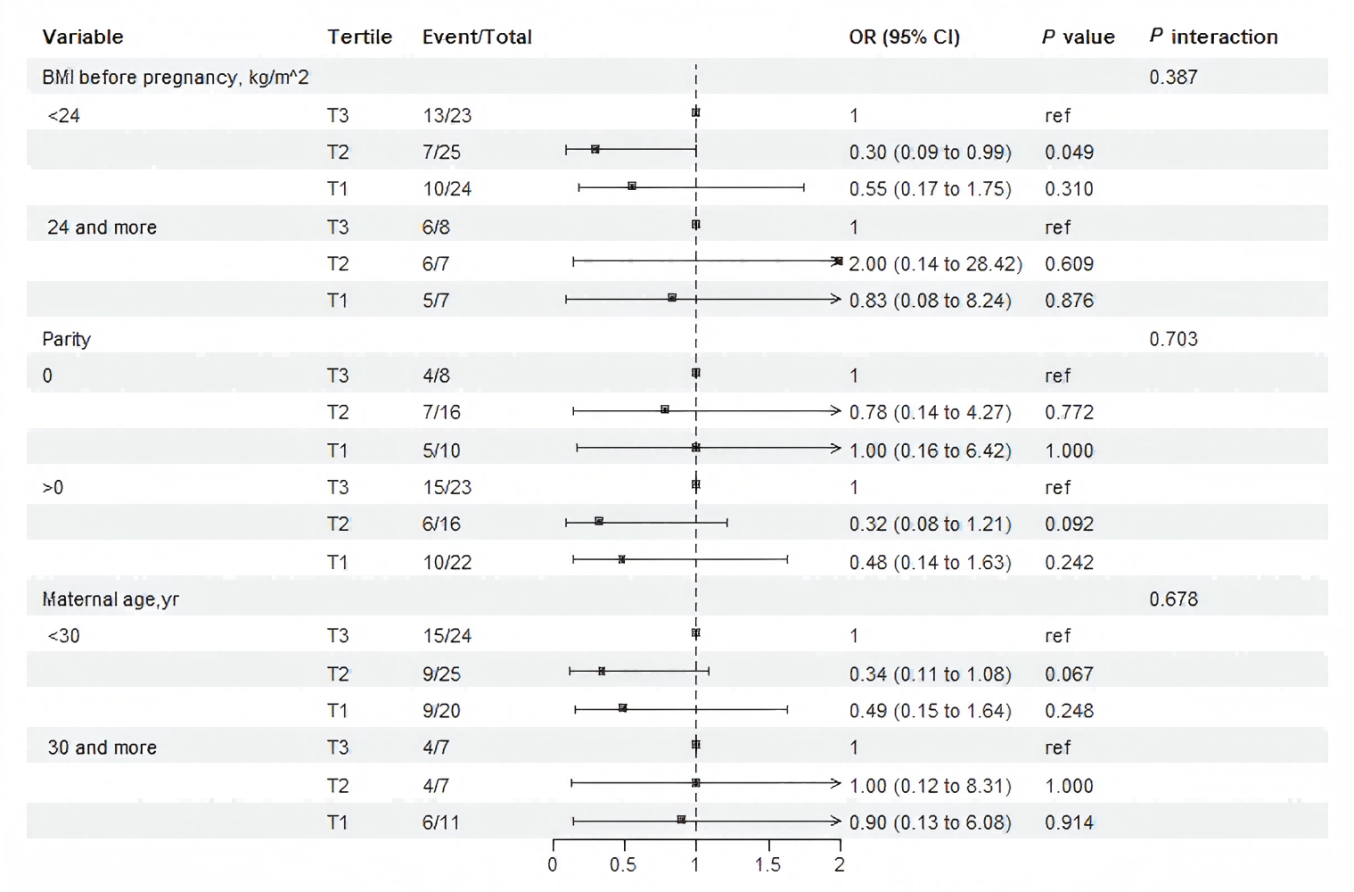
Subgroup and interaction analysis among tertile 1-3 of the protein level of PPAR a in maternal serum and macrosomia occurrence across various subgroups. The analysis was adjusting for gestation in weeks , pre-pregnancy BMI, maternal age, and neonatal sex.

Stratified analysis evaluated whether the associations between *PPAR*α protein and macrosomia varied across prespecified subgroups (Fig. 3). The associations observed in most subgroups was consistent with the main findings of Model 3 (Table 2). No significant interactions were detected (Fig. 3). Notably, in pregnant women with pre-pregnancy BMI <24 kg/m^2^, tertile 2 of maternal serum *PPAR*α protein levels was associated with a 70% reduction in the risk of macrosomia compared with the tertile 3 (*OR*: 0.30, 95% CI [0.09–0.99]; *P* = 0.049).

Sensitivity analysis for *PPAR*α protein levels were broadly consistent with the main findings (Table S3). When restricting the analysis to pregnancies with ≤ 40 weeks’ gestation, tertile 2 remained significantly associated with a reduced risk of macrosomia after covariate adjustment (Model 3: *OR*, 0.20, 95% CI [0.04–0.99]), whereas the corresponding main-model estimate was OR: 0.17 (95% CI [0.03–1.01]) (Table S3, Table 2). Similar results were observed among women without a history of macrosomia and among those with maternal blood samples collected ≤ 13 weeks. Consistent results were also noted after excluding gestational age >40 weeks (Table S3 and Table 2).

### Incremental combination predictive power of serum lncRNA H19-miR-675- PPARα in macrosomia

The basic model included TC, TG, HDL, LDL, maternal age, ethnicity, parity and BMI before pregnancy, and the FBG model further included fasting blood glucose before 20 weeks’ gestation. Given the limited sample size, an exploratory assessment of predictive performance was conducted. Adding the three biomarkers to the basic clinical model did not significantly improve AUC (AUC: 0.799 *vs.* 0.760, *P* = 0.41) (Table 3 and Fig. S3). However, improvements in NRI and IDI—while requiring cautious interpretation due to sample size constraints—suggest that this biomarker combination merits further evaluation in larger cohorts as potential early indicators (Table 3).

**Table 3:** Improvement in discrimination and risk reclassification for macrosomia by adding lncRNA H19, miR675, PPAR a and fasting blood glucose.

Model	AUC (95% CI)	*P* value	NRI (95% CI)	*P* value	IDI (95% CI)	*P* value
Basic model	0.760 (0.665, 0.855)	Ref	Ref		Ref	
+ FBG	0.786 (0.695,0.876)	0.41	0.298 (−0.101, 0.696)	0.143	0.051 (0.007, 0.096)	0.024
+ lncRNA H19, miR675, PPARα protein levels, FBG	0.799 (0.711, 0.888)	0.26	0.511 (0.122, 0.899)	0.010	0.072 (0.020, 0.124)	0.007

**Notes.**

The basic model included TG, TC, HDL, LDL, maternal age, ethnicity, parity, BMI before pregnancy, and maternal education.

The measurements of serum FBG, lncRNA H19, miR675, PPARα protein levels were detected before 20 gestational weeks.

CI confidence interval HDL high density lipoprotein LDL low density lipoprotein AUC area under the receiver operating characteristic curve NRI net reclassification improvement IDI integrated discrimination improvement

## Discussion

We first observed that maternal serum levels of lncRNA H19, miR-675 were not associated with macrosomia, whereas lower serum levels of *PPAR*α protein before mid-gestation were associated with a reduced risk of macrosomia, particularly when the analysis was restricted to pregnancies under 40 weeks’ gestation. In our exploratory analysis, the combination of serum lncRNA H19, miR-675 and *PPAR*α protein level showed preliminary potential as early biomarkers for macrosomia, though this requires further validation. Notably, among singleton pregnant women without obesity (pre-pregnancy BMI <24 kg/m^2^), maternal *PPAR*α protein level before 20 weeks’ gestation were associated with macrosomia risk.

Serum levels of lncRNA H19 has been investigated as a biomarker for various disease progressions (Müller et al., 2019; Petry et al., 2005; Qin et al., 2019; Zhang et al., 2018). In the present study, no differential levels of maternal serum lncRNA H19 and miR-675 were found between the macrosomia and control groups. This aligns with a previous findings showing no significant difference in serum lncRNA H19 expression between healthy pregnancies and those complicated by GDM (Zhang et al., 2018). However, in polycystic ovary syndrome study serum lncRNA H19 emerged as a useful biomarker for the early metabolic dysfunction (Qin et al., 2019). Furthermore, we observed no association between serum lipids and lncRNA H19, which may reflect the limited sample size or indirect links mediated through *PPAR* signaling. In recent intrauterine growth restriction models in rats, the histone modification and methylation of H19 imprinting control region are associated with fetal growth and placental lipid metabolism (Liao et al., 2024). Thus, the potential predictive role of the serum lncRNA H19 for macrosomia risk warrants further investigation.

Pre-pregnancy obesity is a recognized risk factor for fetal overgrowth and macrosomia, and is typically accompanied by elevated serum TC, TG, HDL-C during pregnancy and increased expression of nutrient-sensing signaling pathways such as *PPARs* (Guo et al., 2022; Song et al., 2022). Our results indicate that higher *PPAR*α protein level before 20 weeks’ gestation were associated with increased risk of macrosomia among pregnant women with normal pre-pregnancy BMI. In these women, elevated *PPAR*α protein level may signal abnormal lipid metabolism that could affect placental *PPAR* pathways contributing to macrosomia. This suggests that attention to blood lipid profile and *PPAR*α in early pregnancy should extend beyond obese women. The persistence of this association in women with normal BMI indicates that dysregulated lipid metabolism—reflected by elevated PPARα—may promote fetal overgrowth independently of maternal adiposity. This highlights the importance of metabolic health, not merely weight status, in shaping pregnancy outcomes. Consistent with this perspective, a recent prospective study further highlights the clinical relevance of maternal lipid-glucose metabolism in fetal overgrowth. Firatligil and colleagues (Firatligil et al., 2025) reported that elevated triglyceride-glucose (TyG) index and triglyceride to high-density lipoprotein cholesterol (TG/HDL-C) ratio in late gestation are strongly associated with macrosomia in nulliparous women without GDM, providing specific cut-off values for prediction. Importantly, while their study identified clinically accessible metabolic indices in the third trimester, the association of early-pregnancy PPARα with macrosomia risk observed in our study suggests that the metabolic dysregulation captured by these late-gestation indices may have its origins in, or be reflected by, earlier alterations in key regulatory pathways such as the *PPARs* axis. This temporal link underscores the potential of early-pregnancy molecular markers, like *PPAR*α, for risk stratification before overt metabolic shifts are detectable by conventional lipid-glucose indices. *PPAR*α coordinates de novo lipogenesis and fatty acid production, providing energy reserves during periods of adequate nutrition such as early pregnancy (Dubois et al., 2017). Even in pregnant women with normal BMI, rapid fat accumulation in early and mid-gestation may induce *PPAR*α expression and influence fatty acid transport. Monitoring maternal serum *PPAR*α protein levels may therefore help portray evolving lipid metabolism changes.

Numerous studies have suggested that serum lncRNA H19-miR-675-*PPARs* signatures have the potential to be biomarkers of adverse pregnancy outcomes, women’s diseases, and metabolic diseases. Senousy et al. (2024) reported that serum lncRNA H19 could serve as a biomarker of preeclampsia severity after 20 weeks of pregnancy. Another study showed that low serum LncRNA H19 expression was associated with diminished ovarian reserve based on the results that the low expression of lncRNA H19 in serum is associated with decreased ovarian reserve and increased infertility risk (Xia et al., 2020). In our exploratory analysis, the combined serum biomarkers suggested a possible relationship with macrosomia risk. Although evidence regarding *PPAR*α as a predictor of adverse pregnancy outcomes, remains limited, PPARγ has been linked to polycystic ovary syndrome polycystic ovary syndrome (Chae et al., 2010), dyslipidemia (Matsunaga et al., 2020), and gestational diabetes mellitus (Wang et al., 2019). As reviewed previously, *PPARs* act as a nutrient-sensing signals linking maternal and fetal metabolic syndrome (Tain, Hsu & Chan, 2015). Taken together, serum lncRNA H19-miR-675-*PPAR*α expression levels may represent promising candidate biomarkers for predicting adverse perinatal outcomes, but their clinical utility requires confirmation in larger, prospective studies.

Several limitations should be acknowledged. First, serum lipid levels were not collected longitudinally across early, mid-, and late pregnancy, limiting our ability to characterize lipid dynamics in relation to macrosomia. Second, the modest sample size of this preliminary study constrained statistical power, limited covariate adjustment, and impeded robust validation of the exploratory predictive model. The significant NRI/IDI results, although intriguing, may be unstable in small samples. Larger and more diverse cohorts are essential to validate early-pregnancy *PPAR*α—macrosomia associations, and rigorously evaluate the incremental predictive value and examine longitudinal changes of the H19/miR-675/*PPAR*α axis in both serum and placental tissue. Third, because placenta tissues were not collected, we could not compare the concentration relationship of lncRNA H19, miR-675, and *PPAR*α between maternal blood and placenta. Finally, to preserve the variance required to study lipid metabolism-related biomarkers, pre-pregnancy BMI was adjusted for in multivariable analyses rather than matched at the design stage. However, the decision to adjust for pre-pregnancy BMI rather than match on it represents a methodological trade-off. While this adjustment strategy retains the natural variance essential for biomarker research, BMI remains an incomplete proxy for adiposity and underlying metabolic health. Consequently, residual confounding from unmeasured adipose tissue distribution and function, as well as metabolic factors, cannot be fully excluded, a limitation that is compounded by the modest sample size. Although the association between lower *PPAR*α and reduced macrosomia risk remained significant among non-obese women (BMI <24 kg/m^2^), suggesting an effect independent of obesity, future studies with a larger sample size may consider propensity score matching to further address BMI-related confounding.

## Conclusions

In conclusion, serum lncRNA H19 and miR-675 do not appear to be associated with marosomia risk, while higher serum *PPAR*α protein level before mid-gestation may be associated with an increased risk of macrosomia in singleton pregnancies without GDM. Future studies should expand sample size and track serum and placenta lncRNA H19/miR-675/*PPAR*α expression throughout pregnancy to evaluate their predictive usefulness as early biomarkers for macrosomia.

##  Supplemental Information

10.7717/peerj.20793/supp-1 Supplemental Information 1 Supplementary figures and tables

10.7717/peerj.20793/supp-2 Supplemental Information 2 Dataset

10.7717/peerj.20793/supp-3 Supplemental Information 3 LncRNA H19 CT value

10.7717/peerj.20793/supp-4 Supplemental Information 4 miR-675 cq value

10.7717/peerj.20793/supp-5 Supplemental Information 5 RT-PCR amplification & melt curve

10.7717/peerj.20793/supp-6 Supplemental Information 6 MIQE checklist

10.7717/peerj.20793/supp-7 Supplemental Information 7 STROBE checklist

10.7717/peerj.20793/supp-8 Supplemental Information 8 Codebook

10.7717/peerj.20793/supp-9 Supplemental Information 9 Questionnaire (English)

10.7717/peerj.20793/supp-10 Supplemental Information 10 Questionnaire (Chinese)

## Additional Information and Declarations

### Data Availability

The following information was supplied regarding data availability: The raw data is available in the Supplemental Files.
