# Supplementary material for: Serum lncRNA H19/miR-675 /PPARα expression before middle gestation and their associations with macrosomia risk in singleton pregnancies without gestational diabetes mellitus: a preliminary study
Source: PeerJ. 2026 Feb 16;14:e20793. doi: 10.7717/peerj.20793 (PMC12919319; doi:10.7717/peerj.20793)
Supplement: Supplemental Information 1 [file peerj-14-20793-s001.docx]

Serum lncRNA H19/miR-675/PPARα expression before middle gestation and their associations with macrosomia risk in singleton pregnancies without gestational diabetes mellitus: a preliminary study

Qiu-Yan Yu, MD, PhD^1,*^; Ming-Min Jin, MD^1^; Miao-Miao Ding, MD^1^; Bin-Wei Cheng, MD^1^; Xiao-Xia He, MD, PhD^2^; Xin-Jun Yang, MD, PhD^1,*^

^1^Department of Epidemiology and Health Statistics, School of Public Health, Wenzhou Medical University, Wenzhou, Zhejiang, China

^2^Clinical laboratory, The Affiliated Hospital of Inner Mongolia Medical University, Mongolia, China

**
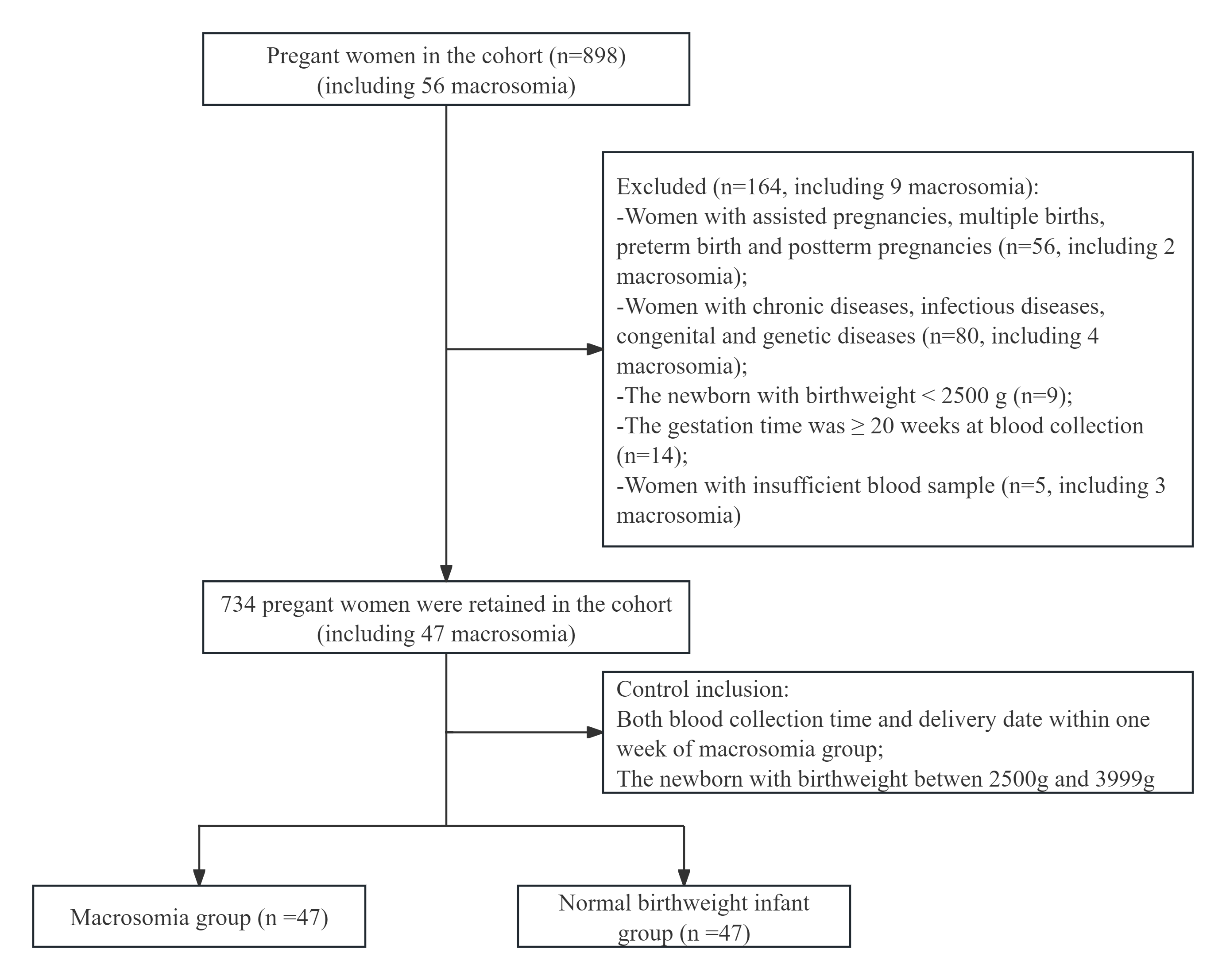
Figure S1** Flow chart of participant screening in the nested case-control study.


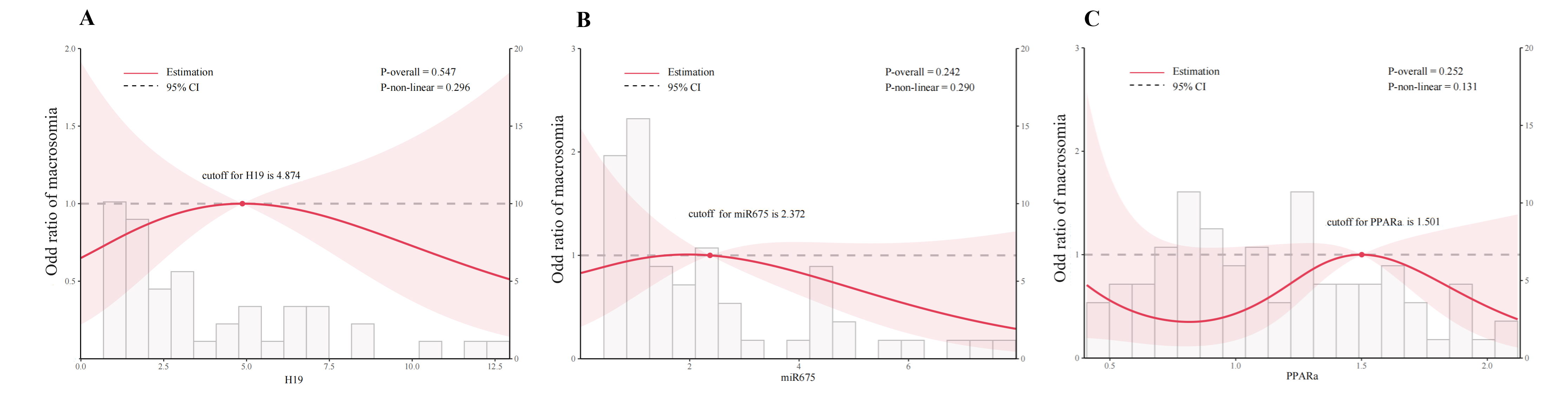


**Figure S2** Restricted cubic spline analysis between maternal serum lncRNA H19, miR-675, the protein level of PPARα and the risk of macrosomia.

A : lncRNA H19; B: miR-675; C: the protein level of PPARα. logistic models are conducted without adjustment of any confounding factors. Odds ratios are indicated solid red lines and 95% *CIs* by shaded areas. The horizontal dotted lines represent the odds ratio of 1 as reference


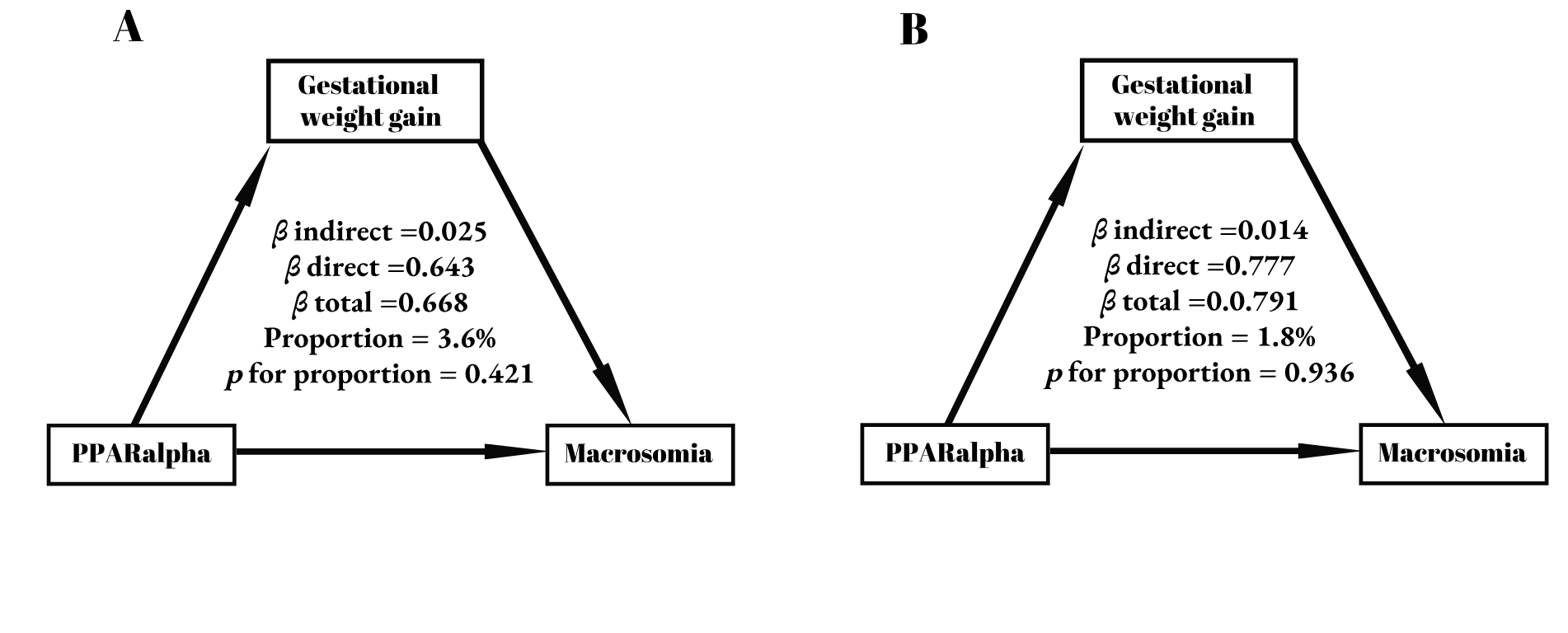


**Figure S3** Mediation effect of gestational weight gain between maternal serum PPARα and macrosomia occurrence

1. :unadjusted by any confounding factors; (B): adjusted by BMI before pregnancy and gestational weeks using conditional logistic models


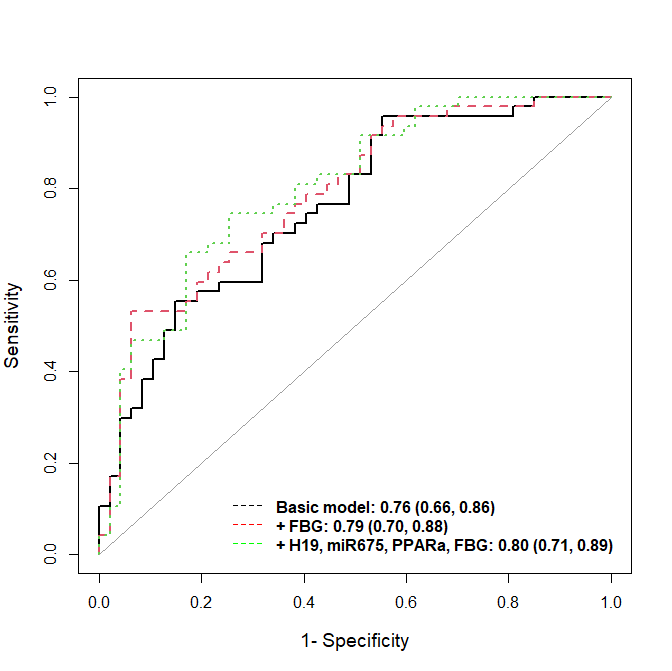


**Figure S4** The receiver operating characteristic curves of maternal serum H19, miR-675, and PPARα as biomarkers to predict macrosomia

The basic model were constructed with TC, TG, HDL, LDL, maternal age, ethnicity, parity, and BMI before pregnancy

**Table S1.** Maternal and neonatal baseline characteristics

| **Characteristics** | Pregnant women (n=898) |
| --- | --- |
| **Maternal age (years)** | 28.00 (25.00, 30.00) |
| **Maternal BMI before pregnancy (kg/m^2^)** | 21.23 (19.54, 23.44) |
| **Education, n (%)** |  |
| ≤ High school | 541 (60.24) |
| > High school | 354 (39.42) |
| Missing | 3 (0.33) |
| **Drinking before pregnancy, n (%)** |  |
| No | 693 (77.17) |
| Yes | 47 (5.23) |
| Missing | 158 (17.59) |
| **Smoking before pregnancy, n (%)** |  |
| No | 732 (81.51) |
| Yes | 14 (1.56) |
| Missing | 152 (16.93) |
| **Exercise during pregnancy, n (%)** |  |
| ≤ 3 times/week | 408 (45.43) |
| 4-6 times/week | 55(6.12) |
| ≥ 7 times/week | 211(23.50) |
| Missing | 224 (24.94) |
| **Sleep duration during pregnancy (h)** | 8.00 (8.00, 9.50) |
| **Gestational weight gain (kg)** | 14.00 (10.58, 17.00) |
| **Blood collection time (weeks)** | 11.00 (9.00, 13.00) |
| **Gestational weeks (weeks)** | 39.00 (38.00, 40.00) |
| **Parity, n (%)** |  |
| Primipara | 357 (39.76) |
| Multipara | 541 (60.24) |
| **Gravidity, n(%)** |  |
| 1 | 261 (29.06) |
| >1 | 637 (70.94) |
| **Mode of delivery, n (%)** |  |
| C-section | 339 (37.75) |
| Vaginal | 557 (62.03) |
| Missing | 2 (0.22) |
| **Infantsex, n(%)** |  |
| Male | 512 (57.02) |
| Female | 386 (42.98) |
| **Infant length (cm)** | 50.00 (50.00, 50.00) |
| **Birthweight (g)** | 3,329.82 ± 442.01 |
| Low birthweight, n(%) | 24 (2.67) |
| Macrosomia, n (%) | 56 (6.24) |

**Table S2.** Comparison analysis of data before and after imputation

| Characteristics | Data without imputation (n = 734) | Data with imputation (n = 734) | P values | SMD |
| --- | --- | --- | --- | --- |
| Maternal age (years) | 27.00 (25.00, 30.00) | 27.00 (25.00, 30.00) | 0.998 | <0.001 |
| Han ethnicity | 683 (93.1) | 682 (93.0) | 1.000 | <0.001 |
| Primipara | 290 (39.5) | 289 (39.4) | 1.000 | 0.002 |
| Receiving ≤ 12 years' education | 437 (59.5) | 437 (59.7) | 0.992 | 0.003 |
| BMI before pregnancy (kg/m^2^) | 21.23 (19.53, 23.41) | 21.23 (19.53, 23.39) | 0.935 | 0.004 |
| Fasting blood glucose (mmol/L) | 4.75 (4.51, 4.95) | 4.75 (4.51, 4.96) | 0.940 | 0.004 |
| Gestational age (weeks) | 39.00 (38.00, 40.00) | 39.00 (38.00, 40.00) | 0.979 | 0.001 |
| Gestational weight gain (kg) | 14.00 (11.00, 17.50) | 14.00 (11.00, 17.20) | 0.914 | 0.007 |
| Vaginal delivery | 475 (64.7) | 475 (64.8) | 1.000 | 0.002 |
| Newborn length (cm) | 50.00 (50.00, 50.00) | 50.00 (50.00, 50.00) | 0.820 | 0.003 |
| < 7000 RMB of family income per month | 391 (53.3) | 299 (52.2) | 0.737 | 0.022 |
| Working during pregnancy | 393 (53.5) | 338 (53.3) | 0.976 | 0.005 |
| Sleep duration during pregnancy | 8.00 (8.00, 9.00) | 8.00 (8.00, 9.00) | 0.744 | 0.016 |
| Drinking before pregnancy | 43 ( 5.9) | 40 ( 5.9) | 1.000 | 0.001 |
| Smoking before pregnancy | 12 ( 1.6) | 12 ( 1.8) | 1.000 | 0.009 |
| < 3 frequencies of physical exercise during pregnancy per week | 443 (60.4) | 363 (59.2) | 0.713 | 0.023 |
| > 7 frequencies of cereals intake per week | 704 (95.9) | 651 (95.7) | 0.973 | 0.009 |
| > 7 frequencies of nuts intake per week | 180 (24.5) | 168 (25.6) | 0.674 | 0.026 |
| > 7 frequencies of meats intake per week | 374 (51.0) | 351 (51.5) | 0.889 | 0.010 |
| > 7 frequencies of seafood intake per week | 202 (27.5) | 192 (28.3) | 0.797 | 0.017 |
| > 7 frequencies of soybean intake per week | 248 (33.8) | 231 (34.0) | 0.971 | 0.005 |
| > 7 frequencies of vegetable intake per week | 569 (77.5) | 525 (76.9) | 0.818 | 0.016 |
| > 1 egg per day | 321 (43.7) | 287 (42.8) | 0.776 | 0.018 |
| > 7 frequencies of fruits intake per week | 601 (81.9) | 549 (81.1) | 0.755 | 0.020 |
| > 7 frequencies of milk and milk products intake per week | 287 (39.1) | 268 (39.5) | 0.930 | 0.008 |
| > 7 frequencies of folic acid intake per week | 693 (94.4) | 580 (94.3) | 1.000 | 0.005 |

Data are show as n (%), mean (SD), or median (lower quartile, upper quartile).

SD= standard deviance. SMD = standardized mean difference.

Multiple Imputation was performed by random forest

**Table S3.** Sensitivity analysis on association of the protein level of PPARα in maternal serum with incidence of macrosomia

| **Variables** | **No. of macrosomia/total** | **Model 1** | | **Model 2** | | **Model 3** | |
| --- | --- | --- | --- | --- | --- | --- | --- |
|  |  | ***cOR* (95%*CI*)** | ***P* value** | ***aOR* (95%*CI*)** | ***P* value** | **a*OR* (95%*CI*)** | ***P* value** |
| **Among neonate-mother pairs ≤ 40 weeks’ gestation** | | | | | | | |
| **Per unit decrease** |  | 0.58 (0.30, 1.12) | 0.104 | 0.64 (0.30, 1.39) | 0.259 | 0.67 (0.30, 1.48) | 0.326 |
| Tertile 1 | 14/30 | 0.28 (0.07, 1.16) | 0.080 | 0.36 (0.07, 1.96) | 0.240 | 0.37 (0.06, 2.14) | 0.268 |
| Tertile 2 | 12/29 | 0.27 (0.07, 1.03) | 0.056 | 0.21 (0.05, 0.97) | 0.045 | 0.20 (0.04, 0.99) | 0.048 |
| Tertile 3 | 15/27 | Reference |  | Reference |  | Reference |  |
| **Among neonate-mother pairs without history of macrosomia** | | | | | | | |
| **Per unit decrease** |  | 0.80 (0.37, 1.72) | 0.566 | 0.95 (0.35, 2.60) | 0.927 | 0.78 (0.20, 3.07) | 0.993 |
| Tertile 1 | 12/26 | 0.58 (0.12, 2.90) | 0.511 | 1.62 (0.15, 18.19) | 0.694 | 1.88 (0.13, 27.93) | 0.955 |
| Tertile 2 | 9/27 | 0.37 (0.09, 1.54) | 0.172 | 0.16 (0.02, 1.15) | 0.068 | 0.14 (0.02, 1.14) | 0.066 |
| Tertile 3 | 14/26 | Reference |  | Reference |  | Reference |  |
| **Among neonate-mother pairs with maternal blood samples collected ≤ 13 weeks’ gestation** | | | | | | | |
| **Per unit decrease** |  | 0.68 (0.34, 1.33) | 0.257 | 0.64 (0.28, 1.45) | 0.286 | 0.64 (0.28,1.45) | 0.285 |
| Tertile 1 | 10/22 | 0.46 (0.12, 1.83) | 0.268 | 0.44 (0.08, 2.45) | 0.351 | 0.44 (0.08, 2.43) | 0.346 |
| Tertile 2 | 10/25 | 0.37 (0.11, 1.29) | 0.118 | 0.27 (0.06, 1.10) | 0.067 | 0.26 (0.06, 1.09) | 0.065 |
| Tertile 3 | 17/27 | Reference |  | Reference |  | Reference |  |

OR (95%CI) was estimated by multivariate conditional logistic regression

Model 2 was conducted with adjusting for pre-pregnancy BMI, maternal age.

Model 3 was conducted with adjusting for pre-pregnancy BMI, maternal age, and neonatal sex.
