# Supplementary figures and images for: Serum lncRNA H19/miR-675 /PPARα expression before middle gestation and their associations with macrosomia risk in singleton pregnancies without gestational diabetes mellitus: a preliminary study
Source: PeerJ. 2026 Feb 16;14:e20793. doi: 10.7717/peerj.20793 (PMC12919319; doi:10.7717/peerj.20793)

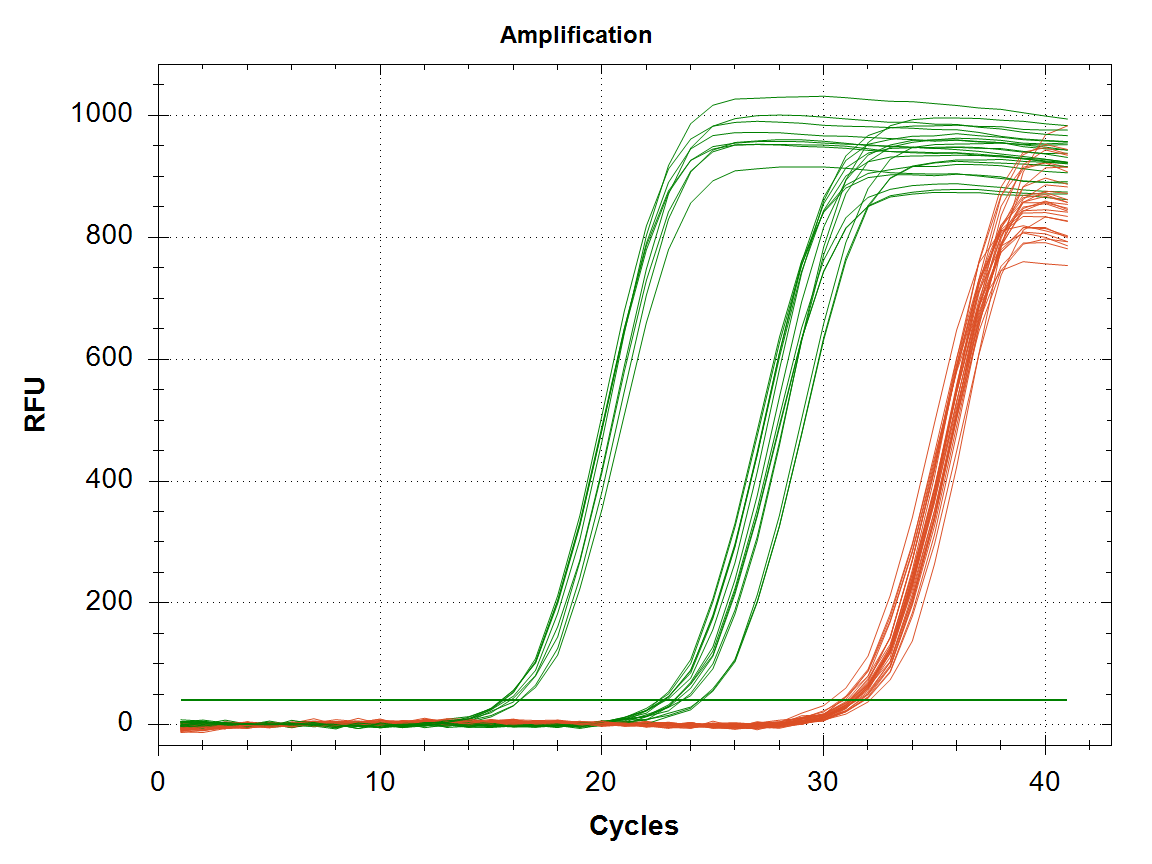

Supplement: Supplemental Information 5 [file peerj-14-20793-s005.zip › RT-PCR╩2╛▌/675└⌐╘÷╟·╧▀.png]

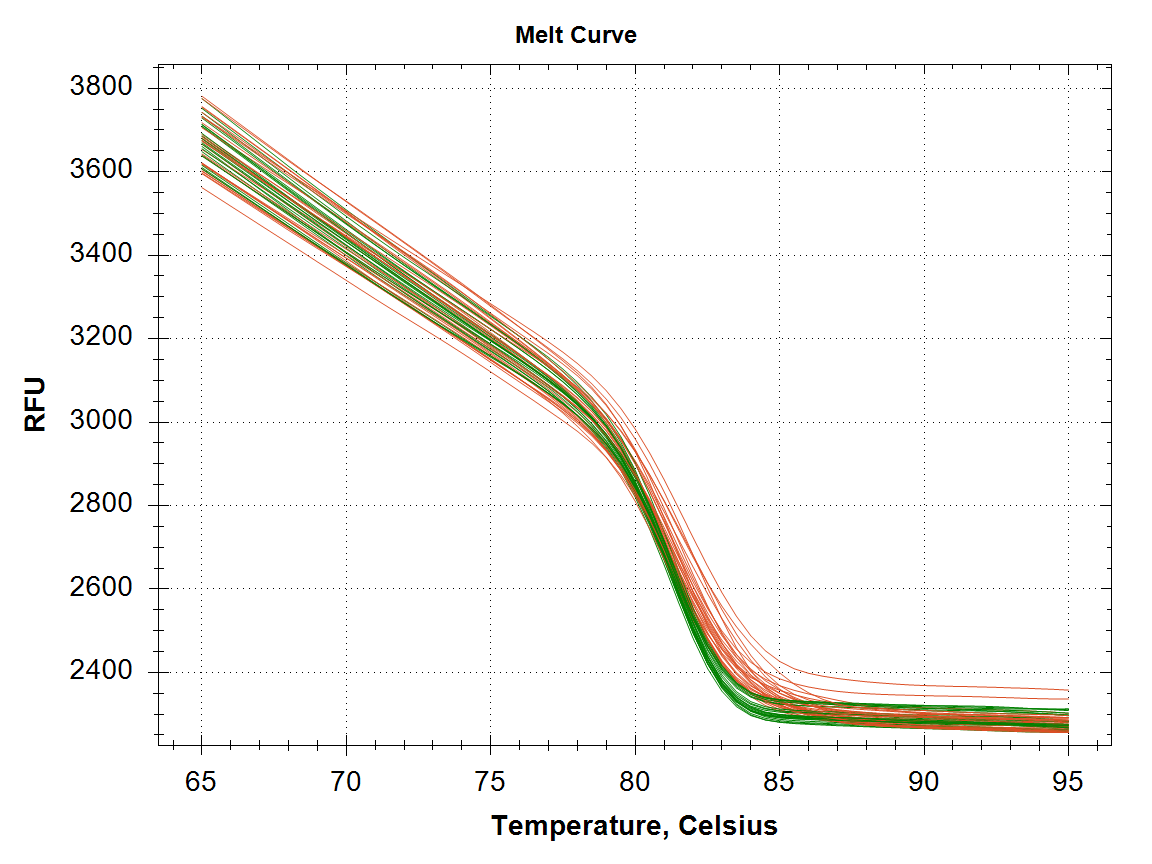

Supplement: Supplemental Information 5 [file peerj-14-20793-s005.zip › RT-PCR╩2╛▌/675╚▄╜Γ╟·╧▀.png]

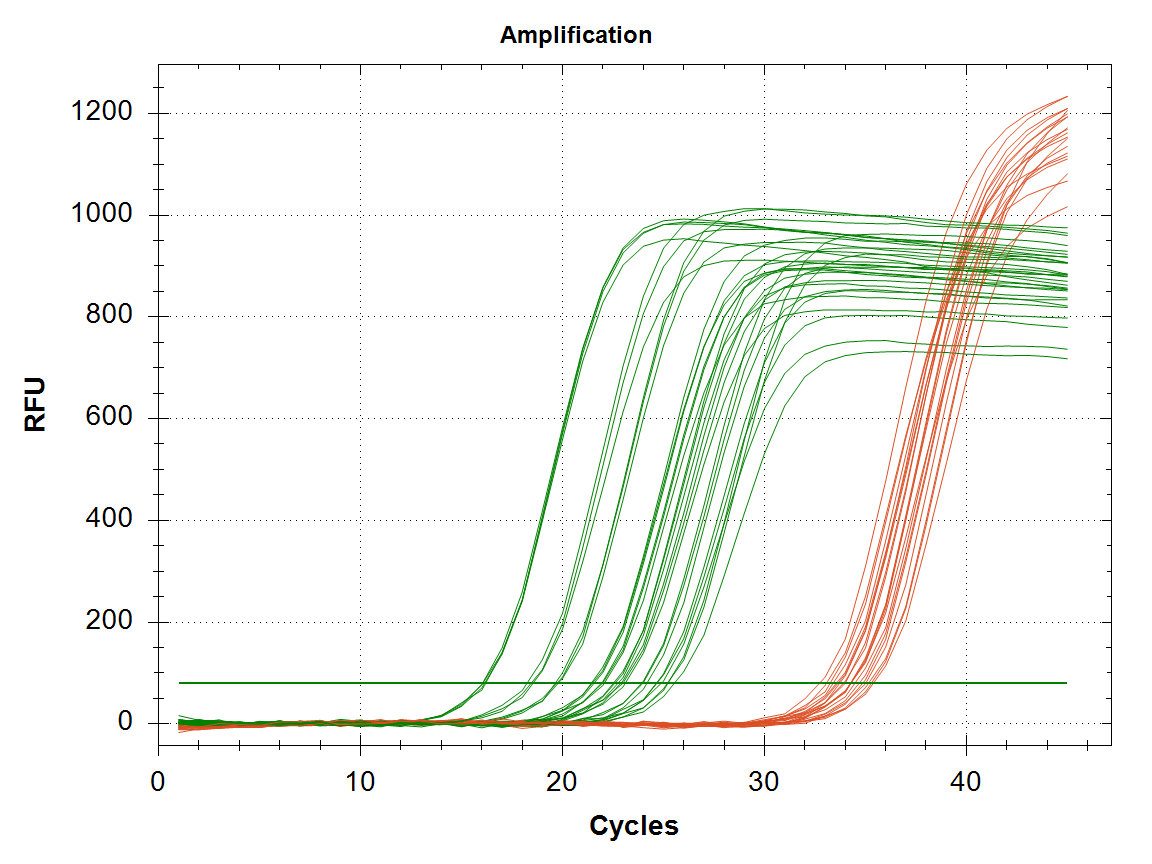

Supplement: Supplemental Information 5 [file peerj-14-20793-s005.zip › RT-PCR╩2╛▌/H19└⌐╘÷╟·╧▀.png]

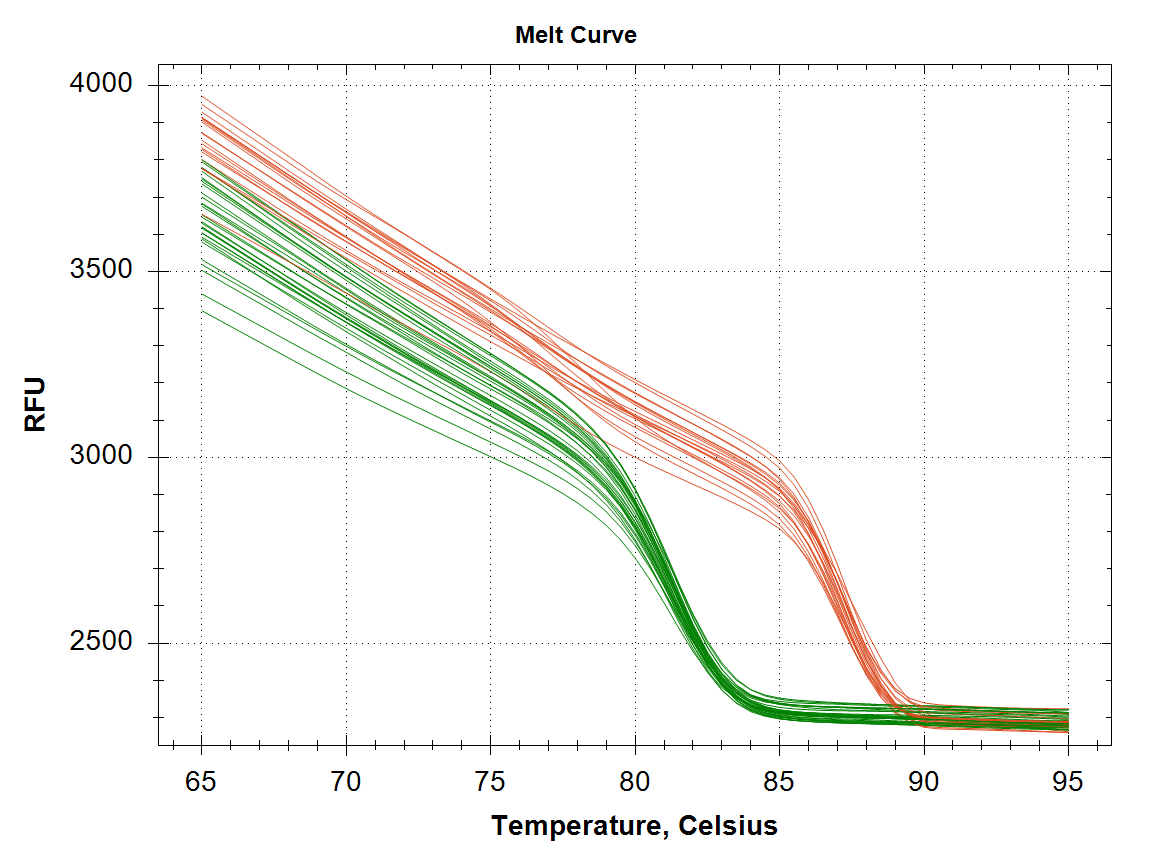

Supplement: Supplemental Information 5 [file peerj-14-20793-s005.zip › RT-PCR╩2╛▌/H19╚▄╜Γ╟·╧▀.png]
