# Supplementary material for: Serum lncRNA H19/miR-675 /PPARα expression before middle gestation and their associations with macrosomia risk in singleton pregnancies without gestational diabetes mellitus: a preliminary study
Source: PeerJ. 2026 Feb 16;14:e20793. doi: 10.7717/peerj.20793 (PMC12919319; doi:10.7717/peerj.20793)
Supplement: Supplemental Information 9 [file peerj-14-20793-s009.docx]

**Pregnancy Health Survey**

Dear Participant,

To understand how maternal health in the first, second, and third trimesters affects fetal development, and to provide a scientific basis for guiding healthy lifestyle choices during pregnancy, we are conducting a study on “The Impact of Maternal Health During Pregnancy on Fetal Outcomes.” Please answer the following questions based on your actual situation. Tick “√” next to the selected option or fill in the blank. Thank you for your cooperation!
(Information provided will be used for scientific research only; all data will be kept confidential.)

Section I. Basic Information

A1. Medical record number: ________; Name: ________; Date of birth (Gregorian calendar): ______ year ______ month.
A2. Pre-pregnancy weight: ______ kg; Height: ______ cm.
A3. Education level:
① Junior high school or below
② Senior high school/technical secondary school
③ College or above

A4. Occupation:
① Housewife
② Civil servant/public institution employee
③ Worker
④ Farmer
⑤ Businessperson
⑥ Other: ____________

A5. Approximate monthly per-capita household income:
① ≤ 1,000 RMB
② 1,000–2,999 RMB
③ 3,000–4,999 RMB
④ ≥ 5,000 RMB

A6. Current residential address: ______ Province, ______ City (County), ______ District, ______ Street. Contact phone: ________.
A7. Was this pregnancy planned?
① No
② Yes
③ Refuse to answer

A8. Did you take oral contraceptives within one year before pregnancy?
① No
② Yes
③ Refuse to answer

A9. Gynecological disorders:
① None
② Yes (specify: __________________)
Were they cured before this pregnancy?
① No  ② Yes

A10. History of miscarriage:
① None
② Yes, spontaneous abortion
③ Yes, induced abortion
④ Refuse to answer

A11. This pregnancy is the ______ gestation; parity ______.
A12. If you already have children, how many? ______ children. For each child:

Child 1: □ son □ daughter Birth weight: ______ jin
Child 2: □ son □ daughter Birth weight: ______ jin
Child 3: □ son □ daughter Birth weight: ______ jin

Section II. Lifestyle During Pregnancy

B1. Current gestational week: ______ weeks; Current weight: ______ kg; Fundal height: ______ cm; Abdominal circumference: ______ cm.
B2. Early-pregnancy symptoms:
B2.1 Any morning sickness?
① No (skip to B3)  ② Yes
B2.2 Medication for morning sickness?
① No  ② Yes

B3. Appetite during pregnancy:
① Poor  ② Average  ③ Very good

B4. Nutritional supplements (calcium, iron, zinc, folic acid, etc.):
① No  ② Yes (specify: __________________)

B5. Cod-liver-oil supplements:
① Never  ② 1–3 times/week  ③ 4–6 times/week  ④ Once daily

B6. Alcohol use:
B6.1 Before pregnancy (≥ once per week for ≥ 6 consecutive months):
① No  ② Yes
B6.2 During pregnancy:
① No  ② Yes

B7. Smoking:
B7.1 Before pregnancy (daily > 1 cigarette for ≥ 3 consecutive months):
① No  ② Yes
B7.2 During pregnancy:
① No  ② Yes

B8. Physical activity:
B8.1 Daily mild/moderate activity (e.g., walking):
① No  ② Yes
B8.2 Average daily duration:
① < 30 min  ② ≥ 30 min

B9. Continue working during pregnancy:
① No  ② Yes

B10. Sleep pattern during pregnancy:
① Regular, ______ hours/day  ② Irregular, average ______ hours/day

Section III. Health Status During Pregnancy

C1. Vaginal bleeding (threatened miscarriage):
① None  ② Yes

C2. Use of tocolytic agents:
① No  ② Yes

C3. Infections during pregnancy:
C3.1 Fever:
① None  ② Yes
C3.2 Common cold:
① None  ② Yes
C3.3 Urinary tract infection:
① None  ② Yes

C4. Medications:
C4.1 Antibiotics:
① No  ② Yes (name: ____________)
C4.2 Antipyretics/analgesics:
① No  ② Yes (name: ____________)
C4.3 Other drugs:
① No  ② Yes (name: ____________)

C5. Exposure to the following:
C5.1 X-ray (radiography, CT, flying, etc.):
① No  ② Yes  ③ Unknown
C5.2 Living in newly renovated house:
① No  ② Yes
C5.3 Passive smoking ≥ once:
① No  ② Yes
C5.4 Long-term exposure to loud noise:
① No  ② Yes

C6. Pre-existing diseases before conception:
① None  ② Yes (specify: ① hypertension ② hepatitis ③ heart disease ④ diabetes ⑤ other: ____________)

C7. Diseases during pregnancy:
① None  ② Yes (specify: ① hypertension ② diabetes or impaired glucose tolerance ③ other: ____________)

Section Diet During Pregnancy

D1. During pregnancy, how would you rate your appetite?

① Poor ② Average ③ Very good

D2. Please recall whether you consumed any of the following foods or nutritional supplements during the past month, and estimate the average portion size and frequency.

Portion sizes: 1 liang = 50 g; 1 cup = 250 mL.

| Foods & Nutritional Supplements | Avg. Portion Size | 2/day | 1/day | 4–6/week | 2–3/week | 1/week | 2–3/month | Rarely/Never |
| --- | --- | --- | --- | --- | --- | --- | --- | --- |
| Rice (liang) |  |  |  |  |  |  |  |  |
| Nuts (peanuts/walnuts, etc.) (liang) |  |  |  |  |  |  |  |  |
| Red meat (pork/beef/mutton) (liang) |  |  |  |  |  |  |  |  |
| Poultry (chicken/duck/goose) (liang) |  |  |  |  |  |  |  |  |
| Organ meats (liang) |  |  |  |  |  |  |  |  |
| Fish (liang) |  |  |  |  |  |  |  |  |
| Seafood (shrimp/crab/shellfish) (liang) |  |  |  |  |  |  |  |  |
| Soy products (tofu, etc.) (liang) |  |  |  |  |  |  |  |  |
| Fresh vegetables (liang) |  |  |  |  |  |  |  |  |
| Pickled vegetables (liang) |  |  |  |  |  |  |  |  |
| Wheat products (buns, bread) (pieces) |  |  |  |  |  |  |  |  |
| Eggs (pieces) |  |  |  |  |  |  |  |  |
| Fresh fruit (pieces) |  |  |  |  |  |  |  |  |
| Soy milk (cups) |  |  |  |  |  |  |  |  |
| Carbonated drinks (cups) |  |  |  |  |  |  |  |  |
| Milk & dairy products (cups) |  |  |  |  |  |  |  |  |
|  |  |  |  |  |  |  |  |  |

D4. Nutritional Supplements (taken as directed)

DHA (often called “brain gold”): ① Yes ② No

Folic acid: ① Yes ② No

Iron: ① Yes ② No

Calcium: ① Yes ② No

Vitamin A/D: ① Yes ② No

Other supplement (name): ___________ ① Yes ② No

Survey member:_____________________ Date:_____________________________________
