# Supplementary material for: Serum lncRNA H19/miR-675 /PPARα expression before middle gestation and their associations with macrosomia risk in singleton pregnancies without gestational diabetes mellitus: a preliminary study
Source: PeerJ. 2026 Feb 16;14:e20793. doi: 10.7717/peerj.20793 (PMC12919319; doi:10.7717/peerj.20793)
Supplement: Supplemental Information 10 [file peerj-14-20793-s010.docx]

**孕妇健康状况调查表**

编号：

您好！

为了解孕妇孕早、中、晚期的健康状况对胎儿的影响，为指导和制定孕妇健康生活方式提供理论依据，特开展此次“孕妇孕期健康状况对胎儿影响”的调查研究。请根据您自身实际情况，在所选答案序号上打“√”，或在空白处填上答案。感谢您的积极配合！（此次问卷填写仅用于科学研究，请放心！）

**一、基本信息**

A1、体检编号： ；姓名： ；/出生年月（公历）： 年 月；

A2、怀孕前体重： kg； 身高： cm；

A3、文化程度: ①初中及以下 ②高中/中专 ③大专、本科及以上

A4、职业：①家庭主妇 ②公务员、事业单位职员 ③工人 ④农民 ⑤经商 ⑥其他：_________

A5、您家庭的人均月收入约：①≤1000元 ②1000元~ ③3000元~ ④≥5000元

A6、家庭现住址： 省 市（县） 区 街 联系电话:

A7、是否为计划好的怀孕: ①否 ②是 ③拒绝回答

A8、怀孕前一年内是否有服用过避孕药：①否 ②是 ③拒绝回答

A9、有无妇科疾患：①无 ②有（若有为 ）;本次怀孕前是否治愈：①否 ②是

A10、是否有过流产经历：①否 ②是，自然流产 ③是，人工流产 ④拒绝回答

A11、本次为第 胎； 第 产；

A12、如已有孩子，有几个孩子？_____个，那么：

第___个孩子是□儿子□女儿（在对应方框内打√），出生体重：______斤 两

第___个孩子是□儿子□女儿（在对应方框内打√），出生体重：______斤 两

第___个孩子是□儿子□女儿（在对应方框内打√），出生体重：______斤 两

**二、孕期生活状况**

B1、目前孕周： 周；现在体重： kg; 宫高： cm；腹围： cm

B2、早孕反应：

B2.1是否有早孕反应: ①否（跳至B3） ②是

B2.2是否服用治疗早孕反应的药物：①否 ②是

B3、孕期食欲：①差 ②一般 ③非常好

B4、孕期是否服用营养补充剂 (包括钙、铁、锌、叶酸等): ①否 ②是：____________

B5、孕期是否服用鱼肝油： ①否 ②1～3次/周 ③4～6次/周 ④1次/天

B6、饮酒史:

B6.1怀孕前是否饮酒（每周至少一次，连续半年以上）：①否 ②是

B6.2孕期是否饮酒：①否 ②是

B7、吸烟史**:**

B7.1怀孕前是否吸烟（每天>1次，连续3个月或以上）：①否 ②是

B7.2 孕期是否吸烟：①否 ②是

B8、孕期运动:

B8.1每天是否有中轻度体力活动(散步等)：①否 ②是

B8.2孕期每天平均活动时间:①＜30 分钟 ②≥30 分钟以上

B9、孕期是否正常工作: ①否 ②是

B10、孕期睡眠是否规律: ①规律， 小时/天 ②不规律，平均 小时/天

**三、孕期健康状况**

调查员: 调查时间: 年 月 日

C1、妊娠期间有无阴道出血（先兆性流产）：①无 ②有

C2、是否曾用过保胎药：①否 ②是

C3、孕期感染史：

C3.1有无发热：①无 ②有；

C3.2有无感冒史：①无 ②有

C3.3有无尿路感染：①无 ②有

C4、孕期用药:

C4.1是否使用抗生素：①否 ②是 若选“是”，药物名称是：

C4.2是否使用解热镇痛药：①否 ②是 若选“是”，药物名称是：

C4.3是否用过其他药物：①否 ②是 若选“是”，药物名称是：

C5、孕期是否接触以下物质:

C5.1 是否被X线（拍片，CT，乘坐飞机等）照射：①否 ②是 ③不知道

C5.2 是否住新装修的房子：①否 ②是

C5.3 是否接受过至少1次被动吸烟：①否 ②是

C5.4 是否长期接受刺耳噪声刺激：①否 ②是

C6、受孕前是否患有如下疾病: ①否 ②是

如果是，请选择: ①高血压 ②肝炎 ③心脏病 ④糖尿病 ⑤其他疾患 (请注明: )

C7、怀孕期间是否患有如下疾病: ①否 ②是

如果是，请选择: ①高血压 ②糖尿病或糖耐量异常 ③其他：

**四 孕期饮食情况**

**D1**怀孕期间，您的食欲如何？
①差 ②一般 ③非常好

D2. 现需要您回忆在过去一个月里，您是否吃过以下食物及营养补充剂，并估计平均食用量和次数（食用量以“两/个/杯”为单位，1两=50 g，1杯=250 mL）。

| 食物及营养补充剂 | 平均每次食用量 | 1天2次 | 1天1次 | 1周4-6次 | 1周2-3次 | 1周1次 | 1月2-3次 | 几乎不吃 |
| --- | --- | --- | --- | --- | --- | --- | --- | --- |
| 米饭（两） |  |  |  |  |  |  |  |  |
| 坚果（花生/核桃等）（两） |  |  |  |  |  |  |  |  |
| 畜肉（猪/牛/羊肉）（两） |  |  |  |  |  |  |  |  |
| 家禽肉（鸡/鸭/鹅）（两） |  |  |  |  |  |  |  |  |
| 内脏类（两） |  |  |  |  |  |  |  |  |
| 鱼（两） |  |  |  |  |  |  |  |  |
| 虾/蟹/贝类等（两） |  |  |  |  |  |  |  |  |
| 豆制品（豆腐等）（两） |  |  |  |  |  |  |  |  |
| 新鲜蔬菜（两） |  |  |  |  |  |  |  |  |
| 腌制蔬菜（两） |  |  |  |  |  |  |  |  |
| 馒头、面包等小麦制品（个） |  |  |  |  |  |  |  |  |
| 蛋类（个） |  |  |  |  |  |  |  |  |
| 新鲜水果（个） |  |  |  |  |  |  |  |  |
| 豆浆（杯） |  |  |  |  |  |  |  |  |
| 碳酸饮料等（杯） |  |  |  |  |  |  |  |  |
| 牛奶及奶制品（杯） |  |  |  |  |  |  |  |  |

D4. 营养补充剂（是否按说明书服用）

DHA（俗称脑黄金）：①是 ②否

叶酸：①是 ②否

铁：①是 ②否

钙：①是 ②否

维生素 A/D：①是 ②否

其他营养补充剂：名称___________ ①是 ②否

调查员: 调查时间: 年 月 日
